# ‘He should feel your pain’: Patient insights on patient–provider communication in Rwanda

**DOI:** 10.4102/phcfm.v10i1.1514

**Published:** 2018-04-16

**Authors:** Vincent K. Cubaka, Michael Schriver, Janvier B. Kayitare, Phil Cotton, Helle T. Maindal, Laetitia Nyirazinyoye, Per Kallestrup

**Affiliations:** 1 School of Medicine and Pharmacy, University of Rwanda, Rwanda; 2 Department of Public Health, Aarhus University, Denmark; 3 School of Public Health, University of Rwanda, Rwanda

## Abstract

**Background:**

Patient–provider communication is an interpersonal interaction between a patient and a health care provider.

**Objective:**

This study explored patients’ communication preferences and perceptions on what factors influence the patient–provider communication in primary health care settings in Rwanda.

**Methods:**

In-depth semi-structured interviews with 15 individuals including 8 with limited literacy. A thematic inductive analysis was used.

**Results:**

Patients valued communication with providers and expressed the need for interacting with caring, empathic providers who can share all the information they want and involve them in their own care. Health literacy and power issues were factors that may influence patient–provider communication. Patients with limited literacy appeared to rely highly on health care providers for making decisions about and managing their health care.

**Conclusion:**

The expressed preferences, including those of patients with limited literacy, aligned well with the patient-centred care model. There were indications of a power imbalance weighing on the provider’s side. Although patients with limited literacy were reliant on providers for decision-making, they were ready to be more involved in the care, suggesting a potential for improved patient involvement even for patients with paternalistic care preferences. These patients’ insights can impact policies and curricula to optimise clinical practice. Generated knowledge will contribute to the indispensable yet underdeveloped field of health communication in sub-Saharan Africa.

**Practice implications:**

Findings call for more inclusion of patient perspectives in the patient–provider encounter. This could require more training of professionals and research on the topic, both in Rwanda and in other regions.

## Introduction

Patient–provider communication (PPC) is a critical component of health care and may influence patients’ health outcomes and well-being directly and indirectly.^1,2^

Patient–provider communication is an interpersonal interaction between a patient and a health care provider (referred to as provider). Exploring opinions of PPC actors is a prerequisite to the quest for optimising PPC, thus improving patient care.^3^ During a clinical encounter, interests and expectations of the provider may differ from that of the patient, making it important to explore patient views; this is well summarised in one publication as: ‘Listen to the patients. They will tell you what they want and need’.^4^ Research on patients’ preferences and perspectives in health care can help develop more effective models and guidelines for practice.^5,6,7^ The more providers and patients are well prepared to communicate, the more effective the interaction will be. Patients with limited health literacy are particularly vulnerable to the interaction, requiring cautious communication efforts.^8,9^

Several theories and models may guide the exploration of PPC,^10,11,12^ one of the most dominant being the ‘patient-centred care’ model.^13,14,15^ It has three core attributes: (1) considering patients’ needs, wants, perspectives and experiences; (2) offering opportunities to patients to provide input into and participate in their care; and (3) enhancing partnership and understanding in the patient–provider relationship.^16^ PPC has been widely studied in Western countries, often from providers’ views,^17^ although more studies are integrating patient experiences and preferences.^2^

In Africa, PPC exploration is scarce, mostly directed towards specific health issues such as HIV and AIDS, TB and maternal health, often with indications of a dearth of patient-centred care.^18,19^ Several reports are found on abusive communication in reproductive health services.^20,21,22^ Patients with HIV and TB reported communication problems such as lack of respect, power imbalance and unclear communication.^23,24,25^ Although outpatient departments (OPD) in primary health care (PHC) receive all patients non-selectively, few studies looked at PPC within general PHC in an African low-income country.^26,27,28^ From our knowledge, no study explored PPC in Rwanda, yet there are several anecdotal critiques in Rwandan media, calling for awareness to develop the relation between healthcare providers and patients.^29,30^

Rwanda is a small landlocked country located in Central East Africa. The population (10.5 million in 2012) is predominantly rural (83%), with a density among the highest in Africa (415 inhabitants per square kilometre).^31^ It has a pyramidal public health system backed by a strong health assurance system (79% of Rwandan households have at least one member covered by health insurance, with 10% service fee paid by the patient for the community-based health insurance) and a network of community health workers who provide home-based care.^32^ Health centres represent the frontline of the Rwandan health system and provide PHC services including ambulatory care, child immunisation, antenatal care, maternity, family planning, HIV care and TB care.^33^ More than 90% of new patient–provider encounters occur in OPD of health centres.^34^ They are led by nurses, most of whom have a secondary school-based nursing education (A2-level).^35^ Furthermore, about 32% of Rwandans aged 15 and above have limited literacy, that is, they are unable to read and write in at least one of the three official languages of the country: Kinyarwanda, English and French.^31^

This study aimed at gaining more insights into patients’ perceptions of their interactions with nurses in PHC settings in Rwanda. The following questions guided the exploration:

- What are patients’ communication preferences?
- What perceived factors may influence communication?

## Methods

### Design

This is an explorative qualitative study using in-depth, semi-structured individual interviews of patients.

### Setting and participants

People above 21 years old who had two or more interactions in the past 12 months with nurses in the OPD of health centres were included, regardless of the health problem. People considered too weak or sick to go through the interview were excluded.

### Sampling

We strategically aimed for around half of informants to be patients who reported themselves as unable to read. We used the term ‘patient with limited literacy’ for this group. This ensured the richness of the data from this potentially vulnerable and often unheard group. During the selection process, we also aimed for gender balance to capture the perspective of both male and female patients. We applied a purposive sampling strategy.^36^ The purpose was to get deep insight into views of patients who had experienced PPC in a consultation room at a health centre in Rwanda. Most informants were approached in the waiting room of two urban and two rural health centres. The interviews were conducted on another agreed day and location to allow time to ‘digest’ the experience of the recent encounter, and for patient convenience. We suggested to meet at our office and gave the alternative to choose another convenient place. Six people were approached in busy public places within 2 km to 5 km of the health centre and interviewed immediately to also include patients without recent contact with a provider. In these cases, the interviews took place in the researchers’ car parked in a quiet place on the side of the road.

We approached 27 people in total, of whom 16 were eligible and willing to participate, and 15 kept the appointment.

### Data collection

The interviewer (J.B.K.), trained in journalism and communication, conducted all interviews and was accompanied by another author (V.K.C.). Interviews were carried out from April to September 2016. Study questions were reviewed by all co-authors, who also contributed to the interview guide, which was translated into Kinyarwanda by the interviewer.

In two pilot interviews with patients with limited literacy, several sentences were incomprehensible or full of truisms without clear relation to the questions asked. This helped develop a simplified version of the interview guide for patients with limited literacy.

Interviews were conducted in Kinyarwanda and were audio recorded, lasting on average 58 min (shortest 24 min, longest 76 min). Interviews were transcribed verbatim in Kinyarwanda, anonymised and translated into English by a professional translator. The interviewer (J.B.K.) control-checked a random page of each English transcript against the Kinyarwanda transcript and the corresponding audio recording and approved the translation.

### Data analysis

A thematic inductive analysis guided by the framework approach was carried out.^37^ MaxQDA 11, a qualitative data analysis software, was used.

Two researchers (V.K.C. and M.S.) familiarised themselves with the 15 transcripts and identified potentially relevant codes. The two researchers together selected, defined and refined emerging key themes to develop a thematic framework. One researcher (V.K.C.) systematically indexed (coded) the text from the 15 transcripts using the agreed thematic framework. Concurrently, data were charted by reorganising it according to the emerging themes and study objectives in a creative and iterative interpretive process, under guidance and discussions with co-authors. Preliminary findings and conclusions were consecutively reviewed by co-authors and disagreements were settled.

### Ethical consideration

Institutional Review Board of the College of Medicine and Health Sciences, University of Rwanda (CHMS/IRB/216/2015 and 324/2016), approved the study. All participants signed an informed consent form prior to their participation. Participants with limited literacy were asked to mark their fingerprints on the informed consent form.

## Results

We present the findings using exemplary quotes, as these elaborate the points we make while conveying a sense of the study field we could not ourselves paraphrase. The purpose was to give direct voice to an often unheard group: patients.

We use the terms ‘she/he’ and ‘her/him’ within quotes to represent a translation of the genderless personal pronouns in the Kinyarwanda language. Gender is specified only when the context allows it. A bracketed with ellipsis […] is used when we omit a portion of the text to simplify the quote, without altering its original meaning as expressed by the patient. Each quote is followed by a person identifier (P1 to P15) in Table 1.

**TABLE 1:** Participant characteristics.

Patient	Age	Sex	Literacy (self-reported)	Recruitment location
P1	38	Female	Unable to read	Health centre
P2	35	Male	Able to read	Health centre
P3	55	Male	Able to read	Health centre
P4	34	Male	Unable to read	Health centre
P5	30	Female	Able to read	Health centre
P6	58	Male	Unable to read	Health centre
P7	29	Female	Unable to read	Health centre
P8	64	Female	Able to read	Health centre
P9	22	Male	Able to read	Health centre
P10	32	Male	Unable to read	Public place
P11	42	Female	Unable to read	Public place
P12	40	Male	Unable to read	Public place
P13	42	Female	Unable to read	Public place
P14	42	Male	Able to read	Public place
P15	45	Female	Able to read	Public place

Fifteen informants were included in the analysis, 7 females (4 self-reported as unable to read) and 8 males (4 self-reported as unable to read). The age range of the informants was 22–64 years (Table 1).

Six main themes emerged from the data:

- patient preferences
- confidentiality and trust
- power issues
- health literacy
- patient satisfaction
- provider lottery and patient choice.

### Patient preferences

Patients brought up a number of wishes and expectations that they believed would improve the interaction with providers. Four subthemes were associated with patient preferences: (1) Welcoming, (2) Attention, (3) Empathy and friendliness and (4) the Right to know.

The first three subthemes can be embedded into the concept of ‘caring behaviour’.

It was highly important for patients that providers receive them in an acceptable way. This is considered the starting point in establishing an instant rapport. A proper greeting may simply be friendly eye contact. Several patients reported that the way they are welcomed may determine the success of the rest of the encounter and a comfortable welcome enables them to share concerns.

‘It doesn’t require me a number of minutes to know whether or not the health care provider is a bad one. It shows through the way he welcomes you in when you enter into the room.’ (P2, male, 35 years old)

Patients requested more attention from providers. This included listening and moving away from distractions and interruptions. The most commonly expressed wish was that informants wanted providers to really listen to them. Patients recognised that this depends on providers:

‘There may be a clinician to whom you start telling an illness but she/he does not understand, cannot list them the way you said. A good clinician is the one […] to whom I explain and she/he listens to me attentively.’ (P1, female, 38 years old)

Patients, including patients with limited literacy, spontaneously requested providers to repeat or rephrase their problems so they felt sure to be understood:

‘When I hear that he talks about everything that I have told him, I understand that he understood me well.’ (P7, female, 29 years old)

Several complained about providers being too hurried to actively listen:

‘There are health care providers who even finish prescribing medications for you before you finish talking and just give you the paper and you go.’ (P3, male, 55 years old)

Some patients suggested providers could ask more about their feelings. Providers should also listen to patients’ feedback. Patients believed their feedback is a way to improve the interactions.

‘The problem that we have is that no meeting is organized for both the health facility staff and the people who live in the health facility catchment area so that we would be able to report the problems that we encounter with. If there was such a meeting, they would solve our problems.’ (P12, male, 40 years old)

Distraction during a consultation was a severe problem to patients:

‘The first thing that he should do is to avoid any distraction while you are conversing.’ (P3, male, 55 years old)

A recurrent distraction and annoyance is the use of telephones:

‘Receiving phone calls or any other things that can distract him, he should stop those things and resume them after he has finished examining me.’ (P7, female, 29 years old)

Writing was often experienced as a distraction:

‘He just looks down onto his paper and keeps writing as if not realizing my presence; but it would be better if he would turn and look at me while he asks me questions and then turn away when he wants to write.’ (P2, male, 35 years old)

In short, informants wanted providers to be ‘present’:

‘I think during the consultation, the clinician should stop other activities like talking to her/his other colleagues, receiving phone calls. She/he should postpone those kinds of activities and examine a patient first. That would be great!’ (P13, female, 42 years old)

Several patients described their preferences for talking without interruptions and experience of being interrupted:

‘Instead of letting you tell them in full details about when and how the illness started, they cut you short […] they do not give patients time to tell them about their illnesses.’ (P3, male, 55 years old)

Time to talk is important because the next steps of the encounter should be based on what the patient said. Sometimes interruptions were experienced as rude:

‘It happens that you start to talk about your illness and the health care provider interrupts you: “your illness is simple”. He makes it simple while it is a burden for you.’ (P9, male, 22 years old)

One informant identified an acceptable interruption as one that deepens understanding:

‘If it is something which is unclear […] he may tell me for example, “hold on a little bit”.’ (P2, male, 35 years old)

It requires certain skills to interrupt in an acceptable manner, with sensitivity:

‘She/he needs to use wisdom, when she/he sees that you are talking too much… There is a way to redirect the conversation.’ (P15, female, 45 years old)

Patients need more than good listeners. They requested empathetic providers who are able to sincerely put themselves in the position of the patient:

‘He should also feel your pain as if the pain was on him.’ (P12, male, 40 years old)

Patients linked empathy with friendliness and ultimately considered it an expression of love:

‘He has to talk to me in a friendly way and I should feel that he has a love for me as a person whom I have come to see.’ (P7, female, 29 years old)

Empathy includes the doctrine of treating patients as one would like to be treated:

‘We would love to see that he is behaving like he is in our shoes and says […] “let me treat this patient as I would like to be treated”.’ (P12, male, 40 years old)

Being friendly was found in almost all interviews and several complained about experiences of unfriendly behaviour:

‘They do not receive you empathetically as someone who is ill, like a suffering person who has come to see a health care provider. They do not care for you as a person who needs them to save your life. There is carelessness.’ (P12, male, 40 years old)

Some thought training could stimulate empathy:

‘The health care providers must be trained so that they know that patients are like friends, close relatives or neighbours.’ (P12, male, 40 years old)

Others seemed less convinced that training was the only answer:

‘He should listen to the patient’s problem whether he was trained or not. But he has to use his heart as a human being in order to interact with the patient.’ (P8, female, 64 years old)

Patients expressed an uncontested desire for clear and precise information from providers about their health. This includes providers’ thoughts, findings and writings and for some also the purpose of examinations:

‘Sometimes, you see her/him writing clinical notes after examining but without explaining to you. Some do not even tell you what you are suffering from, and you go without any explanation, and I think this is a problem!’ (P1, female, 38 years old)

A patient had experienced that requesting further information was a problem:

‘When I asked her “what do the results say? What have they diagnosed?” she rudely asked me “Are you in a position to ask me that? Are you supposed to know it?” She didn’t tell me the illness they had found.’ (P3, male, 55 years old)

Patients also expected guidance, advice and answers. This would require a comprehensive and holistic approach:

‘He should not just give me medications […] otherwise I would return home and live in that place where the illness is originating and fall sick once again. He should go deeply into details and know about my village where I come from, and give me advice of how I have to behave in order to protect myself against that illness.’ (P2, male, 35 years old)

Patients also wished providers would share more information about medication. This would include better information about potential side effects.

‘It’s not a secret, many health care providers make that mistake. He prescribes medications for you, but he doesn’t tell you how to use it.’ (P9, male, 22 years old)

No patients directly raised the issue of consent before an examination, but several wanted explanations:

‘When examining me, she/he should talk to me in a good way explaining what she/he is going to do: if it requires taking off clothes, she/he should say “you will remove these clothes so that I am able to make a physical examination”.’ (P6, male, 58 years old)

Lack of time was not considered an appropriate excuse for lack of information:

‘Maybe it’s because of lack of time, but they should provide information so that patients seek care, with information on how to seek healthcare of course, as some of us are confused of what to do.’ (P13, female, 42 years old)

Information should be given in a simple and clear way:

‘Things that are understandable to people who studied should not be told to someone who didn’t study. If you didn’t study, the health care provider should talk to you in Kinyarwanda and try to get information from you and discover what you value much.’ (P9, male, 22 years old)

### Confidentiality and trust

This section includes issues around confidentiality and privacy, as well as disclosure.

Patients were concerned about confidentiality and privacy of the encounter with providers. Several complained about providers in that regard:

‘It might happen that […] the health care provider is not happy or he is lamenting like “The previous patient was weird” and so forth. He can also for instance warn the next patient, “Don’t behave like the previous patient”. I think that such things should not happen. In short, the next patient should not learn about my interaction with the heath care provider.’ (P2, male, 35 years old)

And:

‘Sometimes you go and take medications and go back home, and when you arrive at home, someone comes and tells you “I am sorry for you, I didn’t know that you had such a problem”.’ (P14, male, 42 years old)

Some complained about consultations with an open door, or concomitant activities in the consultation room disturbing the privacy:

‘In the little room where we are examined, you find other people who cause disorder. That makes the patient uncomfortable […] the consultation rooms should be solely reserved for consultation.’ (P9, male, 22 years old)

Providers’ ability to induce a feeling of trust would have a direct impact on patients’ willingness to disclose their problems:

‘If you do not feel comfortable with her/him, you talk to her/him but you do not tell her/him everything. Sometimes, you become silent as you feel there are things you cannot tell her/him while you went there being very sick.’ (P11, female, 42 years old)

Views of several patients, particularly with limited literacy, indicated a kind of blind or naive trust or faith in providers:

‘When he tells you something, he has a reason why he tells you so […] the health care provider cannot prescribe something bad for you.’ (P7, female, 29 years old)

#### Power issues

This section includes paternalism, power of knowledge, shared decision and patient involvement.

Issues related to paternalism were already mentioned under the theme ‘patient preferences’, which also gave examples of paternalistic practice. One patient compared providers’ power to that of parents and gods, expecting them to act accordingly:

‘Health care providers are like our parents, and they are also like our gods. They should therefore behave like a parent who is conversing with their child.’ (P3, male, 55 years old)

In return, this patient was ready to disclose everything, even follow orders:

‘Because I personally cannot have any problem with the health care provider because I take him like my parent – I cannot hide anything from them. When they tell you “take off your clothes” and you undress, “do like this’” and you do so, there is nothing I can hide from him. I do not have any problem in front of the health care provider, I am ready to do everything that they order me to do without any problem.’ (P3, male, 55 years old)

Many informants felt they had to be obedient towards the provider:

‘I should not have a disagreement with the health care provider because whatever he tells me is what I have to abide by.’ (P10, male, 32 years old)

Most informants regarded themselves below or under providers:

‘My gosh! I have to be below her/him as we are not at the same position. Anyway, it is the reason I come to a health facility so that the clinician - who is above me, who has knowledge, who has learnt/studied something - gives me a piece of advice on how I should do/behave.’ (P6, male, 58 years old)

Patients expressed the view that the quality of care was justly conditioned by the respect patients show providers:

‘Usually, he listens to you; he finds that you have given him the respect and he therefore helps you as somebody who is superior to you, yeah.’ (P5, female, 30 years old)

Some patients, particularly with higher level of education, would see themselves as equal to the provider. But most would highlight the provider as a superior person, who is busy, whose time is precious and who can’t be wrong. Still, many other patients suggested the provider to be humble, and lessen herself or himself to correct the power imbalance for better communication:

‘What should be done to make it better is that the clinician should simplify her/himself, not showing she/he is at the higher position. For me, I am still below but she/he should bear in mind that I’m at the lower position and talk to me humbly without making me worry.’ (P6, male, 58 years old)

Providers were considered to have the answers to the patients’ problems. Informants’ views of providers as superior to them were explained by comparing providers’ knowledge and expertise with their own, and considering their dependency on providers. It was considered natural and unproblematic to be inferior to a provider:

‘The reason why I am under her/him is that there are things he knows, but I don’t know… I only rely on her/him to tell me what to do, or to do something for me.’ (P8, female, 53 years old)

Patients brought up the question of awareness and use of such ‘power of knowledge’ by providers. Several explained they were careful with showing their own knowledge to the provider in order not to be interpreted as a threat, such as:

‘But the one I am familiar with I can tell him that I am having malaria, but the one I am not familiar with I only speak about symptoms, by saying that it started by having fever […] avoiding to show that you know something about it.’ (P15, female, 45 years old)

Most of the patients thought that only the provider should decide because ‘she/he knows’:

‘In the consultation room, the health care provider is the only person who decides everything.’ (P12, male, 40 years old)

Some seemed to have a blind trust in providers’ ability to make decision:

‘She/he cannot actually prescribe medications which would kill me. That’s why I abide by the decisions she/he takes… because she/he knows and she/he has also studied, she/he knows it very well enough, she/he doesn’t learn anything bad that would harm a patient.’ (P2, male, 35 years old)

The concept of making a decision together with the provider was not easily understood:

‘We would probably make bad decisions for ourselves. We could say for example they would give me two pills when they should give me four pills; and that would not be good for me.’ (P3, male, 55 years old)

A middle ground was to have a discussion before the provider decides:

‘She/he can make the final decision, but this should happen after having had a discussion and this happens only when you are comfortable to talk to her/him.’ (P9, male, 22 years old)

Some patients did not experience an opportunity for discussions and hence no opportunity for shared decision:

‘When you are conversing with them, most of the time they ask you something and they write. There is nothing that you discuss, so you cannot say for example “Don’t write that”.’ (P14, male, 42 years old)

While this shows ambiguities in shared decision-making, several patients, including patients with limited literacy, wanted to contribute and actively participate in their care when they had heard examples of its value. During interviews we shared examples of shared decision. Those who were initially opposing would often change their mind based on such an example, reconsidering that perhaps shared decision could be useful.

### Health literacy

Several patients found their health knowledge low and wanted to know more:

‘It is not enough at all, uuh, it’s not enough! […] Sometimes you go and ask yourself “how have I been examined?” You ask yourself why this clinician is not doing like that one, or why I have not been examined like that one.’ (P1, female, 38 years old)

For some patients, low health knowledge would minimise their choice:

‘So, as we seek care while we don’t have knowledge in the medical domain, we take whatever they give us.’ (P8, female, 64 years old)

More knowledge was seen to allow patients to better interact with providers and engage in their own care. However, a patient claimed that explanation was sometimes denied because providers did not believe patients could understand it:

‘If you ask him “What is this device used for?” he tells you “You cannot understand it”. That is difficult […] He could simply tell me “this is used to monitor lungs, this is used to measure blood pressure”.’ (P9, male, 22 years old)

Most informants would describe that their level of education has an impact on their ability to interact with health providers, for instance, the ability to follow an instruction:

‘You may even receive a referral note saying “go there” and you do not know where it is while it was already written on the paper […] because you do not have any level of education.’ (P13, female, 42 years old)

A patient with limited literacy said:

‘When you do not have a certain level of education, you sometimes worry to have a conversation with a clinician… because you seem not knowing anything, you do not read anything. This has an impact.’ (P1, female, 38 years old)

Limited literacy may hamper the ability to explain a health problem to the provider, which may not be easy:

‘We just say superficially our illness, whereas there are other hidden things that we don’t know how to explain. We are actually the ones that should know ourselves! Because it is you, the patient who feel that you have a problem in your body that you have to say, you have to explain your pain, how you feel in your body in a way that the one you are talking to understand it clearly.’ (P8, female, 64 years old)

Several patients with limited literacy tended to overestimate their ability to interact with providers. For instance, when asked about their abilities to converse with providers, most tended to answer very positively, such as:

‘I really feel capable.’ (P1, female, 38 years old)

Whereas literate patients often appeared more reserved about their abilities, such as:

‘My ability to talk to the health care provider is not high enough.’ (P9, male, 22 years old)

Quite often the phrase ‘there is no problem’ would be used by patients with limited literacy when asked to reflect on their own abilities and interaction with providers:

‘There is no problem. There is no challenge there.’ (P8, female, 64 years old)

### Patient satisfaction

It is important that several patients perceived their interaction with providers as generally quite satisfactory. This satisfaction was commonly linked to a request for medication and lab tests:

‘You cannot be pleased by a health care provider who listens to you, but doesn’t give you medications that can help you, even if he has understood your illness.’ (P8, female, 64 years old)

Medication and laboratory tests were the main expectation of patients when seeking care, sometimes valued over good communication:

‘We think that if we have been examined, we must be given medications. If you examine me and explain to me the problem that I have but you don’t give me medications, I will ask myself, “Glad that you have explained to me about my illness; however, am I going to stay like that? Do I have to go home and keep the child there until when?” So, you understand that I will remain with dissatisfaction.’ (P9, male, 22 years old)

While most literate informants requested explanation over medication, several patients with limited literacy had difficulty accepting that medication might not be necessary for a sick child. They seemed to express a complete trust in medication:

‘So, if the health care provider examines the child and tells you the illness that he has found but adds that it is not necessary for the child to take medication […] you wouldn’t understand that explanation as a person who brought the child for medical care. For me, I like the person who immediately gives me the medication that cures my child.’ (P7, female, 29 years old)

### Provider lottery and patient choice

Patients reported differences in the way they are cared for, particularly in the way providers communicate with them. Often, an idea of ‘good’ and ‘bad’ providers was expressed, as well as a perception that one meets ‘a good provider’ by luck:

‘It’s only by luck that you meet with a good health care provider. It is a chance that the Lord gives to you. Then you meet with the one who knows her/his profession and who cares for the human being.’ (P12, male, 40 years old)

We apply the term ‘provider lottery’ to describe this perceived aspect of luck and uncertainty in meeting ‘a good’ provider. The ‘provider lottery’ influenced patients’ health care-seeking behaviour:

‘Sometimes people opt to buy medications at the pharmacy […] because health care providers are different. It all depends on the health care provider that you meet in the consultation room.’ (P3, male, 55 years old)

Although patients may not choose their provider, they can still try to avoid certain individuals with oppressive or judgmental language:

‘She just said “how come that a man like you have ascariasis?” So […] ever since then, whenever I returned to the health centre for care, I always wished to not meet her there. I felt that I would not go in front of her anymore because she looked down on me that time.’ (P3, male, 55 years old)

Most patients did not see any option but going back to the same health facility even if they were unsatisfied last time:

‘I would go back there and accept whoever clinician is available because I cannot stop seeking care.’ (P11, female, 42 years old)

A patient expressed how a bad experience could influence one’s trust in other providers too:

‘Yes, you cannot come back there. From that you make a general assumption that even all other staff are also like him.’ (P8, female, 64 years old)

Several explanations emerged for the differences in providers’ ability to communicate, such as the provider’s personality or mood. Also, work overload would influence care quality on any particular day. Others explained that the lack of communication training would cause differences in care quality:

‘You don’t feel that they were trained in the same way. When you talk to one now and next time talk to the other one you feel that there is a difference.’ (P15, female, 45 years old)

The inability to choose a provider was often described as problematic. One patient directly said:

‘We do not have the right to say, “We want to be seen by such and such health care provider”. If they told patients, “Once a patient arrives at the health facility, they are allowed to choose who will examine them”, this would help patients much more.’ (P3, male, 55 years old)

## Discussion

Patients expressed their communication preferences clearly. They are summarised as a need for a caring provider, for access to more information about their health and health care, and for being involved in their care. These needs are embedded in the patient-centred care model that has impact on health outcomes.^16^ Patients also revealed perceived factors that may affect PPC and thus require further consideration. Patient-centred care is a leading PPC model, yet there are indications that it is not fully conceptualised and applied in our context where the current practice of care may hamper its understanding and implementation.

Despite the relatively low levels of conventional schooling for the majority of our informants, they expressed critical thoughts and deep reflections on PPC. While our data represent narratives of patients, their preferences and needs did not appear as abstract or hypothetical. They rather seemed grounded in and backed by illustrations from their experiences. However, several patients would talk about the kind of behaviour providers should avoid, even if they themselves were reluctant to describe such behaviours as part of their own experiences.

It should be noted that literate patients easily acknowledged the lack of comprehension of a question and gladly asked for clarification before answering. This seldom happened with patients with limited literacy that would often give irrelevant or incomprehensible answers, or feel that everything was perfect. We interpreted this as attempts to hide ignorance or please the interviewer in some way. It may also reflect a Dunning–Kruger effect describing that persons of limited ability may mistakenly assess their ability as greater.^38^ Another explanation could be termed as ‘Nta Kibazo’-effect. Nta Kibazo means ‘No problem’ in Kinyarwanda, ‘Hakuna Matata’ in Swahili, and has equivalents in almost all other African countries. Our informants, particularly with limited literacy, have a background of poverty, being used to daily struggles for maintaining health and life in the family. ‘Nta Kibazo’ could reflect a cultural coping strategy for dealing with adversity ‑ ‘accepting of life’s difficulties’.^39^ This might hide existing problems while generating an impression of ‘comfort’ among people who daily deal with adversity.^40^ While these interpretations require further study, providers need at least to be aware of a potential tendency among patients particularly with limited literacy to underestimate their problems and overestimate their capabilities.

Furthermore, there was a tendency among patients to direct the conversation to satisfaction with tests and medications. This reveals a dilemma to providers: should they simply please patients with tests and treatments as requested, or engage patients through discussions about tests and treatment that may challenge patients’ expectations? The former appears to be an easy and common approach, not least because of busy OPDs and difficulty of making follow-up appointments; however, it may also maintain or cultivate a paternalistic practice ill advised by evidence.^16^

### A caring behaviour

This is probably the most pressing need and is at the core of patient-centred care. Patients’ descriptions of a caring provider follow Mosby’s definition: ‘Actions characteristic of concern for the well-being of a patient, such as sensitivity, comforting, attentive listening, honesty, and non-judgmental acceptance’.^41^ It entails a provider who is friendly, listens, understands and shows interest and empathy. This is someone who ‘feels your pain’, as stressed by several informants. Active listening has been described as the key skill for effective patient-centred care,^16^ and the feeling of not being listened to can damage trust in the provider.^4^ To our informants, trust appeared as a key prerequisite not only for successful encounters but also for successful treatment and recovery. Other studies found trust in providers to impact adherence, patient satisfaction and reported symptom improvement.^42,43^ A recent meta-analysis found that patients who trust their providers tend to report more beneficial health behaviours, less symptoms and higher quality of life.^44^ Patients also wished providers to pay attention to their feelings and react with empathy. Empathy has been linked to better health outcome as well,^2,45,46^ and providers’ attention was a main focus for patients asked to share their views without being restricted by a questionnaire.^4^

### Patients’ rights

Effective communication between patients and providers may be seen as a patient’s right.^47^ This includes the right to knowledge and the right to make choice.^48^

Informants wanted to know the diagnosis and the treatment plan. They believed this would help them participate actively in their care and better take control of their health as also proven by a recent study.^49^ Patients’ perceptions indicate important differences and inconsistency in providers’ communication styles. This inconsistency may pose challenges to effective health communication. It may reflect lack of standardised and effective communication training programmes. This issue triggered some patients to express the need to choose a preferred provider. They would like to interact with the health care providers they are most comfortable with. This is an important aspect of the continuity of care, fundamental in PHC, described as relational continuity.^43,50^ While ideal, this may be challenging in resource-constrained settings, perhaps unfeasible. Contextual factors such as a high turnover and scarcity of providers, as well as inadequate organisation of care, often found in resource-constrained settings, are examples of systemic issues that may make it difficult to honour such requests. However, patients’ rights call for providers’ responsibilities. Providers must be aware of these key requests among patients and seek to accommodate them to the extent possible without generating harm.

### Patient involvement

Prima facie, most patients were hesitant of being involved in their own care but would change their view after we shared examples of the value of patient involvement. Our study shows that patients, particularly with limited literacy, may have strong though modifiable preferences for paternalistic health care providers. ^51^ Perceived power imbalances may inhibit patients’ involvement in their own care, for instance patients hiding their knowledge to avoid problems, and a preference for passivity and reluctance to actively engage with health care providers. Our study indicates that discussing such behaviour may be a means to challenge it, confirmed in other studies.^52,53,54^ This requires a favourable environment for collaboration in which the provider actively invites the patient to participate in the care, while explaining the value of such initiative to gain the full collaboration of the patient. Responding negatively to patients’ need for more participation may maintain patient passivity, hamper patient autonomy and contribute to unimproved outcomes and impaired patient safety.^51^

### Power issues

Patients expressed the asymmetric, hierarchical relationship with providers as naturally explained by the difference in knowledge.^55^ The metaphor of providers as parents or gods captured this well. Such metaphors are not new and often related to paternalistic practice of care.^56^

Rwanda, like most African countries, is often described as a traditional authoritarian society with strongly hierarchical structures, and a history of strict rules with legacies from the colonial period.^57,58^ One could speculate if this oriented the health system towards more provider-centred care models. Limited literacy may amplify the power differential.^59^ This may have patterned the interaction between patients and providers over time and helped condition the patient perceptions uncovered.^3^ Informants perceived the power imbalance as legitimate and necessary, contrasting with common descriptions of the power imbalance reported in the literature, often presented as unwanted.^60^ We found indications of strong dependency among our informants on health care providers, often bordering on blind trust, much like the child without power of choice.^56^ Particularly among patients with limited literacy, trust appeared as a general trust in modern medicine as an ever-present source of cure and solutions to health problems. They also tended to be less critical towards provided health care, often with no interest to understand their own medical condition, left in the hands of the provider. This attitude may increase the risk of providers overlooking symptoms, not getting or sharing all necessary information, or suggesting an inappropriate plan.^61^

Providers should be aware of this issue and would probably benefit from using plain language and asking plenty of open-ended questions, requiring patients to reflect, such as how do you feel about this plan? Patients may also benefit from effective teach-back techniques.^62,63^ Providers need to be aware of the existing power imbalance in their interaction with patients and find ways to reduce its influence on the interaction and relationship.^55^

Feedback was mentioned as a possible way to voice patients’ preferences and concerns. Feedback may help providers to improve their communication skills, as long as they are prepared and ready to receive feedback. This might be challenging if the paternalistic paradigm dominates.

### Implications of this study

This study has provided preliminary insights into PPC in Rwanda. The findings of our study demonstrate once again the importance of valuing patients’ point of view, particularly in a context of limited literacy and health literacy. This calls for more consideration of patients’ perspectives in future explorations of PPC in Rwanda and beyond.

This study also suggests that limited literacy may be linked to a number of health literacy problems and warrants further study on how best to approach this vulnerable group.

The study shows the need for well-designed communication training to improve communication knowledge, skills and attitude to ensure the genuinely caring behaviour expected by patients.

Also, a secure environment should be created for patients to freely express their preferences, feedback and complaints to reach the level of communication excellence they request. To allow patients to give feedback without fear, one approach may be to formalise the feedback, for instance, through an anonymised assessment tool.

### Strengths and limitations

The strategic inclusion of patients with limited literacy gave voice to a marginalised portion of the population, often excluded in research, and allowed access to richer data.^8^

Our findings are drawn from a relatively small sample of 15 patients. While further interviews might have generated more ideas, it was our interpretation that saturation was reached on matters related to our research questions.

This study focused on patients attending outpatient clinics at health centres, and these patients tended to share experiences within the health centre and beyond. We, therefore, believe findings are potentially transferable to PPC in other health services and other care providers in Rwanda.

Limited literacy may reduce informants’ ability to actively engage in the type of open discussion necessary to generate rich qualitative data. This may have limited the quantity and quality of information generated from interviews with patients with limited literacy. We tried to accommodate this by simplifying the question guide and ensuring comfort and privacy during the interviews that were conducted far from the health facilities. Further studies may benefit from exploring the use of pictures and storytelling as means to generate information with this patient group.

## Conclusion

To our knowledge this is the first study exploring patients’ perceptions of their communication with PHC providers in OPD in Rwanda. The participants in this study were able to demonstrate shared and consistent views of the perceived factors that influence effective PPC. Patients’ preferences reflect components of the patient-centred care model and include a need for meeting caring, empathic providers, who can inform them about their health and involve them in health care. Patients with limited literacy appeared to rely highly on health care providers for making decisions about and managing their health care on their behalf. They were, however, ready to be more involved, suggesting a potential for improved patient involvement even for patients with paternalistic care preferences. These findings should be considered while planning services, training health care providers, measuring quality and developing health education strategies to empower patients or simply while discussing issues around PPC. The generated knowledge may help advance PPC and health communication in Rwanda and in sub-Saharan Africa.

## References

[1] Street RL, Makoul G, Arora NK, Epstein RM How does communication heal? Pathways linking clinician-patient communication to health outcomes. Patient Educ Couns. 2009;74:295–301. https://doi.org/10.1016/j.pec.2008.11.015 19150199 10.1016/j.pec.2008.11.015

[2] Street RL How clinician-patient communication contributes to health improvement: Modeling pathways from talk to outcome. Patient Educ Couns. 2013;92:286–291. https://doi.org/10.1016/j.pec.2013.05.004 23746769 10.1016/j.pec.2013.05.004

[3] Schiavo R Health communication: From theory to practice. San Francisco: Jossey-Bass; 2007.

[4] Bensing J, Rimondini M, Visser A What patients want. Patient Educ Couns. 2013;90:287–290. https://doi.org/10.1016/j.pec.2013.01.005 23395286 10.1016/j.pec.2013.01.005

[5] Mulley AG, Trimble C, Elwyn G Stop the silent misdiagnosis: Patients’ preferences matter. BMJ. 2012;345:e6572 https://doi.org/10.1136/bmj.e6572 23137819 10.1136/bmj.e6572

[6] Bastemeijer CM, Voogt L, Van Ewijk JP, Hazelzet JA What do patient values and preferences mean? A taxonomy based on a systematic review of qualitative papers. Patient Educ Couns. 2017;100(5):871–881. https://doi.org/10.1016/j.pec.2016.12.019 28043713 10.1016/j.pec.2016.12.019

[7] Wolfe A Institute of Medicine Report: Crossing the quality chasm: A new health care system for the 21st century. Policy Polit Nurs Pract. 2001;2:233–235. https://doi.org/10.1177/152715440100200312

[8] Volandes AE, Paasche-Orlow MK Health literacy, health inequality and a just healthcare system. Am J Bioeth. 2007;7:5–10. https://doi.org/10.1080/15265160701638520 10.1080/15265160701638520 18027287

[9] Nutbeam D Health literacy as a public health goal: A challenge for contemporary health education and communication strategies into the 21st century. Health Promot Int. 2000;15:259–267. https://doi.org/10.1093/heapro/15.3.259

[10] Bylund CL, Peterson EB, Cameron KA A practitioner’s guide to interpersonal communication theory: An overview and exploration of selected theories. Patient Educ Couns. 2012;87:261–267. https://doi.org/10.1016/j.pec.2011.10.006 22112396 10.1016/j.pec.2011.10.006 PMC3297682

[11] Cheng BSS, Bridges SM, Yiu CKY, McGrath CP A review of communication models and frameworks in a healthcare context. Dent Update. 2015;42:185–186,189–190,193. 26058232 10.12968/denu.2015.42.2.185

[12] Engel GL The need for a new medical model: A challenge for biomedicine. Psychodyn Psychiatry. 2012;40:377 https://doi.org/10.2307/1743658 23002701 10.1521/pdps.2012.40.3.377

[13] Stewart M Towards a global definition of patient centred care. BMJ. 2001;322:444–445. https://doi.org/10.1136/bmj.322.7284.444 11222407 10.1136/bmj.322.7284.444 PMC1119673

[14] Mead N, Bower P Patient-centredness: A conceptual framework and review of the empirical literature. Soc Sci Med. 2000;51:1087–1110. https://doi.org/10.1016/S0277-9536(00)00098-8 11005395 10.1016/s0277-9536(00)00098-8

[15] Epstein RM, Franks P, Fiscella K, et al Measuring patient-centered communication in patient-physician consultations: Theoretical and practical issues. Soc Sci Med. 2005;61:1516–1528. https://doi.org/10.1016/j.socscimed.2005.02.001 16005784 10.1016/j.socscimed.2005.02.001

[16] McWhinney IR Why we need a new clinical method. Scand J Prim Health Care. 1993;11:3–7. https://doi.org/10.3109/02813439308994894 8484077 10.3109/02813439308994894

[17] Jarrett N, Payne S A selective review of the literature on nurse-patient communication: Has the patient’s contribution been neglected? J Adv Nurs. 1995;22:72–78. 7560539 10.1046/j.1365-2648.1995.22010072.x

[18] Ogaji DS, Giles S, Daker-White G, Bower P Systematic review of patients’ views on the quality of primary health care in sub-Saharan Africa. SAGE Open Med. 2015;3:2050312115608338 https://doi.org/10.1177/2050312115608338 27170843 10.1177/2050312115608338 PMC4855308

[19] De Man J, Mayega RW, Sarkar N, et al Patient-centered care and people-centered health systems in sub-Saharan Africa: Why so little of something so badly needed? Int J Pers Cent Med. 2016;6:162–173. https://doi.org/10.5750/IJPCM.V6I3.591

[20] Moyer CA, Adongo PB, Aborigo RA, Hodgson A, Engmann CM ‘They treat you like you are not a human being’: Maltreatment during labour and delivery in rural northern Ghana. Midwifery. 2014;30:262–268. https://doi.org/10.1016/j.midw.2013.05.006 23790959 10.1016/j.midw.2013.05.006

[21] Yakong VN, Rush KL, Bassett-Smith J, Bottorff JL, Robinson C Women’s experiences of seeking reproductive health care in rural Ghana: Challenges for maternal health service utilization. J Adv Nurs. 2010;66:2431–2441. https://doi.org/10.1111/j.1365-2648.2010.05404.x 20735507 10.1111/j.1365-2648.2010.05404.x

[22] Ne U, An G, Aj E, Dst O Client views, perception and satisfaction with immunisation services at Primary Health Care Facilities in Calabar, South-South Nigeria. Asian Pac J Trop Med. 2010;3:298–301. https://doi.org//10.1016/S1995-7645(10)60073-9

[23] Chimbindi N, Bärnighausen ML, Newell T Patient satisfaction with HIV and TB treatment in a public programme in rural KwaZulu-Natal: Evidence from patient-exit interviews. BMC Health Serv Res. 2014;14:32 https://doi.org/10.1186/1472-6963-14-32 24450409 10.1186/1472-6963-14-32 PMC3904687

[24] Gourlay A, Wringe A, Birdthistle I, Mshana G, Michael D, Urassa M ‘It is like that, we didn’t understand each other’: Exploring the influence of patient-provider interactions on prevention of mother-to-child transmission of HIV service use in rural Tanzania. PLoS One. 2014;9:e106325 https://doi.org/10.1371/journal.pone.0106325 25180575 10.1371/journal.pone.0106325 PMC4152246

[25] Kyaddondo D, Wanyenze RK, Kinsman J, Hardon A Home-based HIV counseling and testing: Client experiences and perceptions in Eastern Uganda. BMC Public Health. 2012;12:966 https://doi.org/10.1186/1471-2458-12-966 23146071 10.1186/1471-2458-12-966 PMC3607982

[26] Lau SR, Christensen ST, Andreasen JT Patients’ preferences for patient-centered communication: A survey from an outpatient department in rural Sierra Leone. Patient Educ Couns. 2013;93:312–318. https://doi.org/10.1016/j.pec.2013.06.025 23906648 10.1016/j.pec.2013.06.025

[27] Topp SM, Chipukuma JM A qualitative study of the role of workplace and interpersonal trust in shaping service quality and responsiveness in Zambian primary health centres. Health Policy Plan. 2016;31:192–204. https://doi.org/10.1093/heapol/czv041 25999586 10.1093/heapol/czv041 PMC4748128

[28] Birhanu Z, Assefa T, Woldie M, Morankar S Predictors of perceived empathy among patients visiting primary health-care centers in central Ethiopia. Int J Qual Health Care. 2012;24:161–168. https://doi.org/10.1093/intqhc/mzs001 22302068 10.1093/intqhc/mzs001

[29] Mbonyinshuti JD Customer care is the first dosage, doctors’ boss says [homepage on the Internet]. The New Times. 2013 [cited 2017 March 14]. Available from: http://www.newtimes.co.rw/section/Printer/2013-03-18/63947/

[30] Mbabazi D Midwives: Are labour wards turning into torture chambers? [homepage on the Internet]. The New Times. 2015 [cited 2017 March 14]. Available from: http://www.newtimes.co.rw/section/article/2015-03-12/186810/

[31] National Institute of Statistics of Rwanda (NISR) [Rwanda], Ministry of Health (MOH) [Rwanda] 2012 Rwanda 4th Population and Housing Census [homepage on the Internet]. 2012 [cited 2017 March 14]. Available from: http://microdata.statistics.gov.rw/index.php/catalog/65/download/508

[32] Ministry of Health [Rwanda] National Community Health strategic plan 2013–2018 [homepage on the Internet]. 2013 [cited 2017 March 15]:84. Available from: http://www.moh.gov.rw/fileadmin/templates/CHD_Docs/CHD-Strategic_plan.pdf

[33] Ministry of Health (MOH) [Rwanda] Service packages for health facilities at different levels of service delivery [homepage on the Internet]. 2011 [cited 2017 March 15]. Available from: http://www.moh.gov.rw/fileadmin/templates/Clinical/SERVICE-PACKAGES-FOR-HEALTH-FACILITIES-AT-DIFFERENT-LEVELS-OF-SERVICE-DELIVERY-last-version.pdf

[34] National Institute of Statistics of Rwanda (NISR) Statistical yearbook, 2016 edition (SYB2016) [homepage on the Internet]. 2016 [cited 2017 March 14]. Available from: http://www.statistics.gov.rw/publication/statistical-yearbook-2016

[35] Ministry of Health [Rwanda] Human resources for health strategic plan 2011–2016. Kigali: Ministry of Health 2011.

[36] Reid S, Mash B African primary care research: Qualitative interviewing in primary care. Afr J Prim Health Care Fam Med. 2014;6:1–6. 10.4102/phcfm.v6i1.632 PMC4502895 26245436

[37] Ritchie J, Spencer E Qualitative data analysis for applied policy research. In: Bryman A, Burgess RG, editors Analysing qualitative data. London: Routledge, 1993; p. 173–194.

[38] Kruger J, Dunning D Unskilled and unaware of it: How difficulties in recognizing one’s own incompetence lead to inflated self-assessments. J Pers Soc Psychol. 1999;77:1121–1134. https://doi.org/10.1037/0022-3514.77.6.1121 10626367 10.1037//0022-3514.77.6.1121

[39] Gardenswartz L, Rowe A Managing diversity: A complete desk reference & planning guide [homepage on the Internet]. 3rd ed. Society for Human Resource Management; 2010 [cited 2017 April 08]. Available from: http://www.barnesandnoble.com/w/managing-diversity-lee-gardenswartz/1101365127#productInfoTabs

[40] Barbato CA, Perse EM Interpersonal communication motives and the life position of elders. Commun Res. 1992;19:516–531. https://doi.org/10.1177/009365092019004007

[41] Mosby’s medical dictionary 9th ed. St. Louis: Mosby Elsevier; 2009.

[42] Thom DH, Kravitz RL, Bell RA, Krupat E, Azari R Patient trust in the physician: Relationship to patient requests. Fam Pract. 2002;19:476–483. https://doi.org/10.1046/j.1369-6513.2000.00091.x 12356698 10.1093/fampra/19.5.476

[43] Baker R, Mainous AG, Gray DP, Love MM Exploration of the relationship between continuity, trust in regular doctors and patient satisfaction with consultations with family doctors. Scand J Prim Health Care. 2003;21:27–32. https://doi.org/10.1080/0283430310000528 12718457 10.1080/0283430310000528

[44] Birkhäuer J, Gaab J, Kossowsky J, et al Trust in the health care professional and health outcome: A meta-analysis. PLoS One. 2017;12:e0170988 https://doi.org/10.1371/journal.pone.0170988 28170443 10.1371/journal.pone.0170988 PMC5295692

[45] Markham F, Hojat M, Louis DZ, Markham FW Physicians ’ empathy and clinical outcomes for diabetic patients physicians’ empathy and clinical outcomes. Acad Med. 2011;86:359–364. https://doi.org/10.1097/ACM.0b013e3182086fe1 21248604 10.1097/ACM.0b013e3182086fe1

[46] Derksen F, Olde Hartman TC, Van Dijk A, Plouvier A, Bensing J, Lagro-Janssen A Consequences of the presence and absence of empathy during consultations in primary care: A focus group study with patients. Patient Educ Couns. 2017;100(5):987–993. https://doi.org/10.1016/j.pec.2016.12.003 27989493 10.1016/j.pec.2016.12.003

[47] Gostin L, Hodge JG Jr., Valentine N, Nygren-Krug H The domains of health responsiveness – A human rights analysis. Geneva: World Health Organisation; 2003.

[48] Murray CJL, Frenk J A framework for assessing the performance of health systems. Bull World Health Organ. 2000;78:717–731. https://doi.org/10.1590/S0042-96862000000600004 10916909 PMC2560787

[49] Sacks RM, Greene J, Hibbard J, Overton V, Parrotta CD Does patient activation predict the course of type 2 diabetes? A longitudinal study. Patient Educ Couns. 2017;100(7):1268–1275. https://doi.org/10.1016/j.pec.2017.01.014 28159442 10.1016/j.pec.2017.01.014

[50] Haggerty JL, Reid RJ, Freeman GK, Starfield BH, Adair CE, McKendry R Continuity of care: A multidisciplinary review. Br Med J. 2003;327:1219–1221. https://doi.org/10.1136/bmj.327.7425.1219 14630762 10.1136/bmj.327.7425.1219 PMC274066

[51] Longtin Y, Sax H, Leape LL, Sheridan SE, Donaldson L, Pittet D Patient participation: Current knowledge and applicability to patient safety. Mayo Clin Proc. 2010;85:53–62. https://doi.org/10.4065/mcp.2009.0248 20042562 10.4065/mcp.2009.0248 PMC2800278

[52] Moreau A, Carol L, Dedianne MC, et al What perceptions do patients have of decision making (DM)? Toward an integrative patient-centered care model. A qualitative study using focus-group interviews. Patient Educ Couns. 2012;87:206–211. https://doi.org/10.1016/j.pec.2011.08.010 21903355 10.1016/j.pec.2011.08.010

[53] Charles C, Gafni A, Whelan T Shared decision-making in the medical encounter: What does what does it mean? (Or it takes at least two to tango). Soc Sci Med. 1997;44:681–692. 9032835 10.1016/s0277-9536(96)00221-3

[54] Joseph-Williams N, Elwyn G, Edwards A Knowledge is not power for patients: A systematic review and thematic synthesis of patient-reported barriers and facilitators to shared decision making. Patient Educ Couns. 2014;94:291–309. https://doi.org/10.1016/j.pec.2013.10.031 24305642 10.1016/j.pec.2013.10.031

[55] Kettunen T, Poskiparta M, Gerlander M Nurse-patient power relationship: Preliminary evidence of patients’ power messages. Patient Educ Couns. 2002;47:101–113. https://doi.org/10.1016/S0738-3991(01)00179-3 12191533 10.1016/s0738-3991(01)00179-3

[56] Rees CE, Knight LV, Wilkinson CE Doctors being up there and we being down here: A metaphorical analysis of talk about student/doctor-patient relationships. Soc Sci Med. 2007;65:725–737. https://doi.org/10.1016/j.socscimed.2007.03.044 17493722 10.1016/j.socscimed.2007.03.044

[57] Van Dormael M La médecine coloniale, ou la tradition exogène de la médecine moderne dans le Tiers Monde. Stud Health Serv Organ Policy [serial online]. 1997 [cited 2017 March 15];1 Available from: http://dspace.itg.be/bitstream/handle/10390/4995/1997shso0001.pdf?sequence=1

[58] Waller JE Becoming evil: How ordinary people commit genocide and mass killing. 2nd ed. New York: Oxford University Press; 2002.

[59] Paasche-Orlow MK, Schillinger D, Greene SM, Wagner EH How health care systems can begin to address the challenge of limited literacy. J Gen Intern Med. 2006;21:884–887. https://doi.org/10.1111/j.1525-1497.2006.00544.x 16881952 10.1111/j.1525-1497.2006.00544.x PMC1831564

[60] Dickerson SS, Brennan PF The internet as a catalyst for shifting power in provider-patient relationships. Nurs Outlook. 2002;50:195–203. https://doi.org/10.1067/mno.2002.128446 12386654 10.1067/mno.2002.128446

[61] Paasche-Orlow MK, Wolf MS The causal pathways linking health literacy to health outcomes. Am J Health Behav. 2007;31(Suppl 1):S19–S26. https://doi.org/10.5555/ajhb.2007.31.supp.S19 17931132 10.5555/ajhb.2007.31.supp.S19

[62] Tamura-Lis W Teach-back for quality education and patient safety. Urol Nurs. 2013;33:267–298. https://doi.org/10.7257/1053-816X.2013.33.6.267 24592519

[63] Jager AJ, Wynia MK Who gets a teach-back? Patient-reported incidence of experiencing a teach-back. J Health Commun. 2012;17:294–302. https://doi.org/10.1080/10810730.2012.712624 10.1080/10810730.2012.712624 23030577

